# Editorial: T Cell Regulation by the Environment

**DOI:** 10.3389/fimmu.2015.00229

**Published:** 2015-05-13

**Authors:** David A. Hafler, Anne L. Astier

**Affiliations:** ^1^School of Medicine, Yale University, New Haven, CT, USA; ^2^MRC Centre for Inflammation Research, University of Edinburgh, UK

**Keywords:** T cells, environment, metabolism, microbiome, vitamin D, regulatory T cells, pathogens

T cell responses are initiated by ligation of their cognate T cell receptor by MHC loaded with antigenic peptide, but their response is carefully controlled by a myriad of environmental cues, including co-activation receptors, cytokines, nutrients, growth factors, local oxygen levels, salt concentrations, and microbiome. The complexity of the integration of signals received by T cells is only beginning to be fully understood (1). This research topic in T cell biology aims at highlighting some of the latest research on intrinsic and extrinsic signals regulating T cell responses. The ebook contains 10 articles that encompass key pathways that modulate T cell function and discuss how T cells coordinate their response to environmental cues.

One of the first components regulating T cell activation is the expression and subsequent activation of surface receptors. Notably, T cell activation is governed by the co-activation of the TCR and of co-stimulatory or co-inhibitory molecules (2, 3). Expression and activation of co-inhibitory molecules, such as CTLA-4 and PD1 play a key part in turning off effector responses and the balance of expression of co-stimulatory and co-inhibitory receptors needs to be tightly regulated to ensure a proper level of T cell activation (4, 5). The review by Schneider and Rudd nicely illustrates how expression of the co-inhibitory molecule CTLA-4 is regulated, and how that affects T cell activation (6).

In recent years, the importance of cellular metabolism in the activation of immune cells, in particular T cells, has been reported. T cell activation requires a change in cellular metabolism to face increased energetic demand. This is an exciting area of research showing that not only changes in metabolism are necessary for cell activation but that they are also actively involved in regulating T cell function and differentiation. This is discussed in several reviews. Craig Byersdorfer focuses on the role of fatty acid oxidation for T cell functions, and notably on its role in graft versus host disease (7). Ramsay and Cantrell discuss the importance of glucose metabolism for T cell function and highlight the role of hypoxia-inducible factor alpha and mTOR in coordinating the responses to the environmental cues (8). They also summarize the importance of the microbiome in regulating T cells, and the key role of the aryl hydrocarbon receptor in sensing microbes. Clovis Palmer and collaborators review glucose metabolism in T cells and also discuss how HIV infection modulates T cell metabolism (9). One of the central questions is how do T cells integrate the multitude of signals received? In their comprehensive review, Chapman and Chi discuss the central role of mTOR in the integration of the many signals received by the T cells that ultimately shape their response (10).

Another related aspect of the topic is the impact of infection on T cells, whereby pathogens control the host response, mostly to their advantage, and are able to switch T cell responses to favor their own survival. A study by the group of Francisca Mutapi describes a novel mechanism by which helminth infection downregulates T cell activation, by lowering the level of CD3zeta chain in infected individuals (11). A review from Zaunders further illustrates how HIV infection affects the balance of the various T cell subsets and promotes regulatory T cells and T helper follicular cells (12).

Diet can also influence immune homeostasis, with recent studies showing, for instance, how salt can affect Th differentiation (13). The effects of vitamins and their metabolites on T cells are also well described. Colleen Hayes’ group summarizes the latest data on the effects of Vitamin D on T cell responses, and how this can be modulated in autoimmune diseases (14). A review by Leving’s group focuses more particularly on the environmental factors that affect regulatory T cells. This includes cytokines, vitamin A and vitamin D, metabolism, and microbiome (15).

Finally, can we therapeutically manipulate the environmental milieu to modulate T cell responses in humans? The study by the group of David Klatzmann highlights the effects of IL-2 in modulating T cell responses in type 1 diabetes (16). While the functions of regulatory CD4+ Tregs have been described years ago, the characterization of CD8+ Tregs is more recent. The authors notably report how injection of low doses of IL-2 modulates the levels of CD8+ Tregs *in vivo*, in both mice and in patients with type 1 diabetes, following their numbers and phenotype. This study illustrates how exogenous cytokines can modulate *in vivo* T cell responses, which may prove beneficial for autoimmune diseases.

We hope that this compilation of reviews and research that provides an overview on environmental regulators of T cells will give the readers a flavor on the latest development in T cell biology. We would also like to thank all the contributors to this topic and the reviewers for their time in making this ebook possible.

## Conflict of Interest Statement

The authors declare that the research was conducted in the absence of any commercial or financial relationships that could be construed as a potential conflict of interest.
